# Effects of Static Meditation Practice on Blood Lipid Levels: A Systematic Review and Meta-Analysis

**DOI:** 10.3390/healthcare12060655

**Published:** 2024-03-14

**Authors:** Michele Antonelli, Davide Donelli, Filippo Luca Gurgoglione, Davide Lazzeroni, Geza Halasz, Giampaolo Niccoli

**Affiliations:** 1 Department of Public Health, AUSL-IRCCS of Reggio Emilia, 42122 Reggio Emilia, Italy; 2 Cardiology Unit, University Hospital of Parma, 43126 Parma, Italy; 3 Prevention and Rehabilitation Unit, IRCCS Fondazione Don Gnocchi, 43100 Parma, Italy; 4 Department of Cardiothoracic and Vascular Medicine and Surgery, Division of Cardiology A.O. San Camillo-Forlanini, 00152 Rome, Italy

**Keywords:** meditation, cholesterol, triglycerides, blood lipids, cardiovascular prevention, health promotion, review

## Abstract

This review aims to delineate the potential impact of static meditation practice on cholesterol and triglyceride levels. PubMed, EMBASE, Web of Science, Cochrane Library, and Google Scholar were systematically screened up until December 2023 to identify pertinent studies. After searching the scientific literature, 16 clinical studies (11 trials and 5 observational experiments) met the criteria for inclusion, involving a total of 1147 participants. In general, Ayurvedic-based meditation techniques were predominantly associated with lower total cholesterol levels, mindfulness-based techniques demonstrated benefits in both total cholesterol and triglyceride levels, and Eastern meditation techniques with spiritual origins were primarily linked to improved serum concentrations of HDL cholesterol. Study participants mostly engaged in meditation on a daily basis, often practicing it once or even twice a day, spanning a duration ranging from a few weeks to several months. The meta-analysis shows an association between meditation practice in healthy or sub-healthy adults and reduced cholesterol levels, with an average decrease of approximately −14 mg/dL (MD = −13.91 [−23.35; −4.47] mg/dL; *p* = 0.02), alongside favorable and even more pronounced impacts on triglyceride levels (MD = −32.56 [−48.44; −16.68] mg/dL; *p* < 0.01). In summary, regular engagement in static meditation practices can be associated with lower triglyceride and, to a lesser extent, cholesterol levels. Further studies on the topic are recommended to better investigate the relationship between meditation practice and physiological parameters.

## 1. Introduction

### 1.1. Background

Over recent years, meditation has evolved from a spiritual pursuit to a valuable complementary approach in various health contexts [1,2]. Scientific studies initially focused on the physiological effects of meditation, but, as interest grew, the lack of a consistent operational definition became evident in publications [3]. In 1971, Wallace et al. firstly proposed the term “wakeful hypometabolic physiologic state”, emphasizing the physiological characteristics of a meditative status without detailing the method [4]. Other early definitions included that of Goleman, who described meditation as “a consistent attempt to reach a specific attention position” [5], and the analysis by Craven, who stressed the different components of meditative practices, namely psychophysical relaxation, concentration, an altered state of consciousness, and a self-observation attitude [6]. In 1998, the Department of Psychobiology at the Universidade Federal de São Paulo initiated systematic studies on meditation, addressing the challenge of defining meditation operationally: they introduced a comprehensive concept, asserting that meditation should encompass a distinct and clearly defined technique that incorporates both physical and “logical” relaxation, involving abstaining from analysis, judgment, and the creation of expectations during the process [3]. Furthermore, it must be a self-induced state, utilizing focus skills to ensure autonomy from the instructor. This definition aims to standardize meditation procedures, acknowledging the subtle interplay between the “anchor” (self-focus skill) and “logical relaxation” essential to meditation, thus contributing to a more precise understanding of this multifaceted practice [3]. Recent efforts to further articulate a definition of meditation have once more emphasized key concepts such as relaxation, attention, and mindfulness, underlining its inherent qualities of inducing a peaceful state of serenity and encouraging a focused engagement with the immediate present [7]. Many meditation techniques can be included in this definition, ranging from mindfulness and mantra/sound-based practices to those centered on breathing or specific body movements, such as yoga or qigong [7].

The hypothesis that stress may elevate blood lipid levels can be understood through the relationship between sympathetic nervous system activity and lipid metabolism [8]. Stress often triggers heightened sympathetic activity, evident in individuals who are overweight or insulin-resistant, characterized by elevated arterial norepinephrine and non-esterified fatty acids [9]. This increased sympathetic nervous system activity correlates with specific lipid profiles, including complex lipids like ceramides, sphingomyelins, phosphatidylcholines, and gangliosides, independently of insulin resistance. Consequently, stress-induced sympathetic activation could potentially modify lipid levels by promoting lipid accumulation through mechanisms such as decreased lipolysis, increased lipogenesis, and the impaired transportation of free fatty acids, largely due to catecholamine resistance encountered during the dynamic phase of fat accumulation [10]. Notably, interventions like exercise and forest bathing have been recognized for their ability to mitigate catecholamine resistance, thereby lowering systemic sympathetic activity, reducing circulating catecholamine levels, and enhancing β-adrenergic receptor signaling, offering a multifaceted approach to managing stress-induced alterations in lipid metabolism [11,12].

In general, the regular practice of meditation has been associated with demonstrated benefits for several aspects of the individual’s well-being: from a psychological point of view, it can contribute to stress reduction, strengthen emotional resilience, hone cognitive functions, improve sleep quality, and mitigate symptoms of anxiety and depression [13,14,15,16,17]; on the physiological front, meditation can lower blood pressure, help manage pain, boost immune defenses, reduce inflammation, balance stress hormone levels, and even slow down some aging processes [18,19,20,21,22,23,24]. In particular, it is well demonstrated that stress reduction can positively contribute to cardiac health [25], and that meditation practices can play a useful integrative role in cardiovascular prevention by positively modulating different risk factors, including psychosocial stress, high blood pressure, smoking habits, alcohol abuse, elevated blood glucose, and lipid levels [26,27,28,29]. Movement-based meditation techniques, such as yoga, are associated with significantly reduced total cholesterol (−10.31 mg/dL on average) and triglyceride (−13.50 mg/dL on average) levels [30]. Similar trends have been demonstrated for qigong, a traditional Chinese practice characterized by moving meditation [31,32,33]. However, to our knowledge, no systematic synthesis has been made so far to analyze the effects of static meditation techniques on blood lipid levels.

### 1.2. Research Aim

This article aims to delineate the potential impact of static meditation practice on blood lipid (cholesterol and triglyceride) levels, exploring its implications as a means to modulate cardiovascular risk factors.

## 2. Methods

### 2.1. Eligibility Criteria

This study was structured as a systematic analysis of the existing literature, and its findings are presented following the guidelines outlined in the PRISMA statement [34]. The review plan was registered on “searchRxiv” with a unique DOI: 10.1079/searchRxiv.2024.00436.

We included pertinent clinical investigations that examined the impact of meditation practices on blood lipid levels, without imposing any restrictions on publication dates. To ensure the reliability and strength of our selection criteria, studies needed to be accessible in the English language or, at the very least, include an English abstract or summary. Additionally, the chosen studies had to be formally published in peer-reviewed journals as original research articles.

The following PICOS criteria were applied for article inclusion in this review:

- P (population): individuals categorized as either healthy subjects or patients with specific disorders, with a particular emphasis on those with cardiometabolic risk factors or diseases.
- I (intervention): all forms of static meditation practiced for varying durations. Studies wherein the meditation intervention was combined with other approaches, such as dietary recommendations, massage, or aerobic physical exercises, were excluded to prevent confounding factors.
- C (comparison): the comparison category encompassed any type, including scenarios with no control.
- O (outcomes): significant alterations in blood lipid levels, covering total cholesterol, HDL cholesterol (HDL-C), LDL cholesterol (LDL-C), and triglycerides.
- S (study design): all clinical investigations comprising both trials and observational studies. Laboratory experiments conducted in vitro or in vivo with animal or cell models were intentionally excluded from the primary search.

### 2.2. Information Sources

To attain an optimal methodological combination of scientific databases [35], a systematic screening of PubMed, EMBASE, Web of Science, Cochrane Library, and Google Scholar was conducted to identify pertinent studies. The search encompassed data from the inception of these databases up until December 2023.

### 2.3. Search Strategy

The search strategies used for each scientific database (and the number of research items retrieved) were the following:

PubMed (*n* = 74): (meditation [Title/Abstract] OR meditative [Title/Abstract] OR “mind-body intervention*” [Title/Abstract] OR “mind-body technique*” [Title/Abstract] OR zen [Title/Abstract] OR “yoga nidra” [Title/Abstract] OR “kundalini yoga” [Title/Abstract] OR “relaxing exercise*” [Title/Abstract] OR “relaxing technique*” [Title/Abstract]) AND (cholesterol [Title/Abstract] OR lipoprotein* [Title/Abstract] OR “lipo-protein*” [Title/Abstract] OR HDL [Title/Abstract] OR LDL [Title/Abstract] OR “blood lipid*” [Title/Abstract] OR “blood fat *” [Title/Abstract] OR triglyceride* [Title/Abstract]).

EMBASE (*n* = 119): (meditation: ti,ab,kw OR meditative: ti,ab,kw OR ‘mind-body intervention*’: ti,ab,kw OR ‘mind-body technique*’: ti,ab,kw OR zen: ti,ab,kw OR ‘yoga nidra’: ti,ab,kw OR ‘kundalini yoga’: ti,ab,kw OR ‘relaxing exercise*’: ti,ab,kw OR ‘relaxing technique*’: ti,ab,kw) AND (cholesterol: ti,ab,kw OR lipoprotein*: ti,ab,kw OR ‘lipo-protein*’: ti,ab,kw OR hdl: ti,ab,kw OR ldl: ti,ab,kw OR ‘blood lipid*’: ti,ab,kw OR ‘blood fat*’: ti,ab,kw OR triglyceride*: ti,ab,kw).

Web of Science (*n* = 309): (TS = (meditation) OR TS = (meditative) OR TS = (mind-body intervention*) OR TS = “mind-body technique*” OR TS = (zen) OR TS = (yoga nidra) OR TS = (kundalini yoga) OR TS = (relaxing exercise*) OR TS = (relaxing technique*)) AND (TS = (cholesterol) OR TS = (lipoprotein*) OR TS = (lipo-protein*) OR TS = (blood lipid*) OR TS = (blood fat*) OR TS = (triglyceride*)).

Cochrane Library (*n* = 43): (“Meditation”) AND (“Cholesterol” OR “Triglycerides”) in Title Abstract Keyword (word variations were searched).

Google Scholar (limited to the first 100 results): “Meditation” AND (“Cholesterol” OR “Triglycerides”) AND “Trial”. The search in this database was limited to the first 100 results to achieve the best accuracy.

### 2.4. Selection Process

One researcher (M.A.) initially assessed all materials acquired through the database search, focusing on titles and abstracts. Following this, a second investigator (D.D.) examined articles that fulfilled the above-mentioned criteria for inclusion. This two-tiered assessment process was structured to guarantee a proper selection of pertinent studies for subsequent analysis.

### 2.5. Data Collection Process

One investigator (M.A.) manually compiled information from studies meeting the inclusion criteria using an Excel spreadsheet. Concurrently, a second researcher (D.D.) executed a random verification process to validate the accuracy and completeness of the collected data. In the case of discrepancies, the other authors were consulted.

### 2.6. Data Items and Effect Measures

The essential data components extracted from the studies incorporated into the review included participant demographics, specific research methodologies, relevant details about the intervention and its comparison, as well as the documented outcomes.

### 2.7. Study Risk of Bias Assessment

Each eligible Randomized Controlled Trial (RCT) underwent evaluation utilizing the Cochrane risk of bias tool [36,37]. Six domains were assessed, including appropriate random sequence generation and allocation concealment, the handling of incomplete outcome data (potentially leading to attrition bias), the evaluation of selective reporting, and scrutiny of other potential sources of bias. In particular, the blinding of study participants was considered irrelevant, given the inherent challenge of concealing meditation from those actively practicing it. Similarly, the blinding of the outcome assessment was also not considered a key area, as the outcome of interest (blood lipid levels) is an objective laboratory measurement that cannot be influenced by the research staff. Each domain was subject to judgment as at a low, unclear, or high risk of bias based on information disclosed in the full-text report of each study. The overall risk of bias was considered low when all domains exhibited a low risk of bias, unclear if one domain was assessed with unclear risk, and high if at least two domains were categorized as unclear risk or if one domain was designated as high risk. This evaluation of the risk of bias, performed by two investigators (M.A., D.D.), played a useful role in informing the analysis of the review. The other researchers helped in the case of disagreements between the first two authors.

The quality evaluation of studies, other than RCTs, was conducted using specific assessment tools provided by the National Institutes of Health (NIH). Specifically, the assessment frameworks used included the quality assessment tools for controlled intervention studies, pre–post studies, and case–control studies, each selected based on the type of study being evaluated. For more detailed information on these assessment tools, the NIH’s dedicated webpage can be consulted at https://www.nhlbi.nih.gov/health-topics/study-quality-assessment-tools (accessed on 9 March 2024).

### 2.8. Synthesis Methods

The most relevant findings were briefly summarized and subsequently underwent discussion to derive a qualitative synthesis. Following this, a quantitative synthesis involving selected RCTs was executed, utilizing the most updated version of the “metaHUN” online software (version 2.0) [38]. The inclusion of articles in the quantitative synthesis adhered to the following PICOS criteria:

- P (population): patients afflicted with chronic diseases or healthy individuals (considering only per-protocol and not intention-to-treat study populations).
- I (intervention): all forms of static meditation.
- C (comparison): no meditation.
- O (outcomes): end-of-study (or change-from-baseline) blood values of total cholesterol, HDL-C, LDL-C, and triglycerides. When necessary, data were converted from mmol/L to mg/dL.
- S (study design): Only RCTs were considered. Non-randomized trials and pre–post studies were excluded from the quantitative synthesis, due to a potential bias in outcomes [39,40].

The process of extracting data from trials incorporated into the quantitative synthesis entailed acquiring mean values and standard deviations. The meta-analysis utilized the raw Mean Difference (MD) as the summary statistic, adopting a random effects model to address heterogeneity among the studies [41]. Statistical heterogeneity was quantified with I^2^ and significance was conventionally set at *p* < 0.05. A leave-one-out analysis was employed to assess the consistency of the meta-analytical results by excluding studies identified as having a high risk of bias. It was decided to assess publication bias with funnel plots only if the number of studies exceeded ten, as recommended by the Cochrane recommendations [42].

## 3. Results

### 3.1. Qualitative Synthesis of the Available Evidence

**Figure 1:**
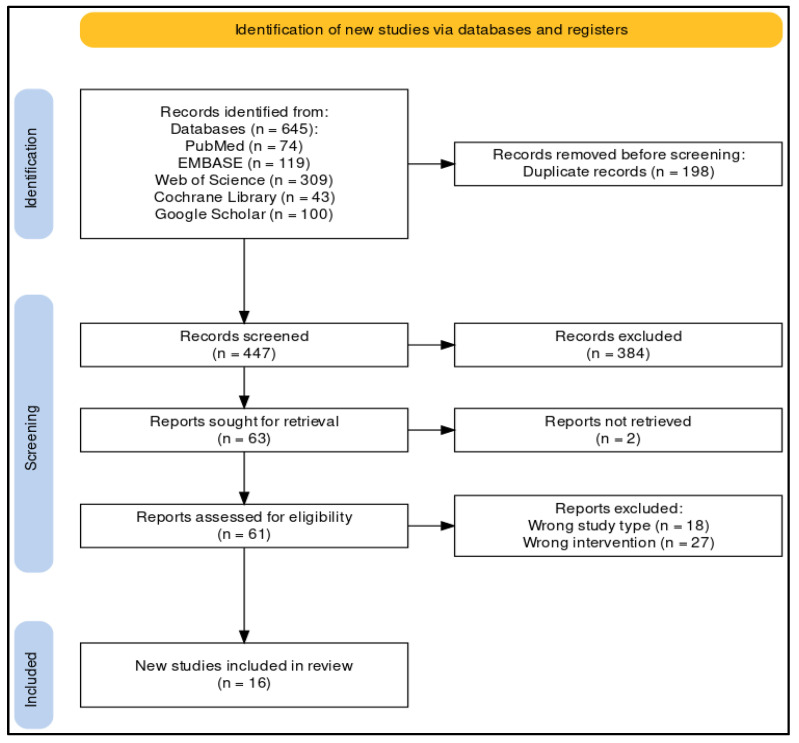
PRISMA flowchart of the article selection process [34,60].

The search strategy yielded a total of 645 results, and, after the article screening process (Figure 1), 16 studies were included in this literature review [32,43,44,45,46,47,48,49,50,51,52,53,54,55,56,57]. The full-text version of two articles was not retrievable online, and these studies were therefore excluded from this research [58,59]. Table 1 summarizes the most relevant characteristics of the studies about the effects of static meditation practice on cholesterol and triglyceride levels.

As shown in Table 1, the studies included in this systematic review were six RCTs [43,44,45,46,47,48], four controlled trials without randomization [32,49,50,51], a pre–post experiment [52], and five observational studies [53,54,55,56,57].

Among the RCTs, the study sample ranged from 35 to 103 units (median: 53), and, in all but one study [46], the participants were adult patients with cardiovascular risk factors (hypertension, diabetes mellitus, metabolic syndrome) or subjects with a history of cardiac ischemic disease. Meditation sessions occurred with a frequency ranging from a minimum of twice a week for several weeks to as much as twice a day over the course of up to one year (refer to Table 1 for specifics). The control group consisted of individuals not engaged in any meditation program. Although the observed benefits were primarily evident in terms of lowered triglyceride and total cholesterol levels [44,45,46], the variations between the intervention and control groups did not reach statistical significance in all instances (see Table 1 for additional details). In particular, the studies reporting statistically significant results favoring the intervention focused on a demographic consisting of younger individuals who were generally in good health or sub-healthy, with high levels of stress but no previous history of heart diseases [44,45,46].

In the context of Non-Randomized Controlled Trials, participants, predominantly healthy or sub-healthy adults, varied from 17 to 76 (median: 27). Meditation was a daily practice, with some individuals engaging even twice a day, spanning periods from several weeks to over a year (refer to Table 1 for comprehensive details). The comparison group abstained from any form of meditation, except in one instance where the control group engaged in relaxation with closed eyes without practicing meditation [50]. Across all studies, the intervention consistently led to significant improvements in the participants’ cholesterol levels compared to those of the control group.

In the only study employing a pre–post design within this review [52], the regular practice of mindfulness-based meditation daily for a duration of two months demonstrated a correlation with enhanced cholesterol levels in healthy participants. Additionally, it was linked to decreased triglyceride levels, observed in both healthy individuals and patients with depression.

All eligible observational studies featured a case–control design, including participant numbers ranging from as few as 49 to as many as 252 (median: 105). The study population predominantly comprised healthy individuals of both genders, with a focus on women in the postmenopausal period [56,57] (see Table 1 for additional details). Cases were selected from individuals predominantly possessing a background in meditation practice, whereas controls were chosen from subjects who had never participated in mind–body practices but maintained similar lifestyle habits as the cases. Notably, the cases exhibited significantly lower cholesterol levels, although triglyceride levels did not display a significant difference between the two groups (see Table 1 for further information).

Table 2 provides a synthesis of study outcomes concerning the impact of static meditation techniques on blood lipid levels. In particular, the examined meditation techniques included Ayurvedic-based practices like Transcendental or Yoga Meditation (excluding body movements) [43,45,46,47,50,51,55,57], mindfulness [44,49,52], and meditation techniques deeply rooted in Eastern spirituality (such as Korean, Zen, Buddhist, and Tibetan practices) [32,48,53,54,56]. The results outlined in Table 2 indicate that Ayurvedic-based meditation techniques were predominantly associated with lower total cholesterol levels, mindfulness-based techniques demonstrated benefits in both total cholesterol and triglyceride levels, and Eastern meditation techniques with spiritual origins were primarily linked to improved serum concentrations of HDL cholesterol. While the majority of studies supported the benefits of Ayurvedic-based practices, scientific evidence was comparatively more limited for other forms of meditation.

The study quality assessment of the RCTs included in this review is reported in Table 3 [43,44,45,46,47,48]. The blinding of participants and the concealment of the outcome assessment were not deemed relevant for the reasons explained in the Methods section (see Section 2.7). From a methodological point of view, the most critical issues were poor information about the randomization procedure, allocation concealment, and significant dropout rates in some instances; however, the overall risk of bias was generally low (see Table 3 for additional details).

The most notable limitations observed in studies, other than RCTs, included inadequate details regarding the power analysis of the study, the small size of the sample group, no specifics of the treatment protocol, and the methods used for allocation concealment. Further details are provided in Table 4.

### 3.2. Meta-Analysis of Quantitative Results

**Figure 2:**
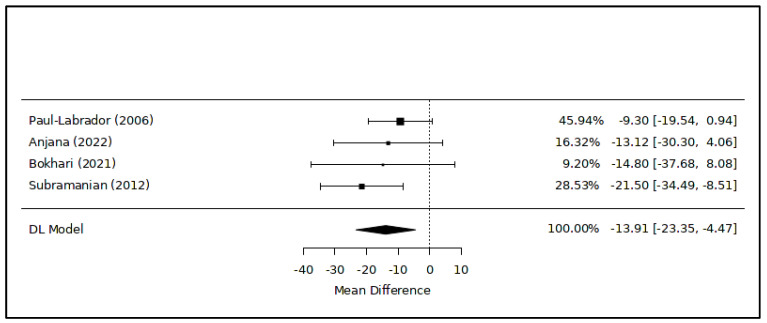
Meta-analysis of variations in total cholesterol levels associated with meditation. Figure caption: The first column lists the names of the first authors and the publication dates of the included studies [43,45,46,47]. The graphical segments represent the effect sizes of individual studies, while the diamond symbolizes the combined results of the quantitative synthesis. The contribution of each study, indicated as its weight, is detailed in the column on the right.

The meta-analysis examining alterations in total cholesterol levels linked to meditation comprised four RCTs [43,45,46,47] (Figure 2), revealing a significant difference between the intervention and control groups. Specifically, individuals engaged in regular static meditation exhibited lower cholesterol levels by approximately 14 mg/dL compared to non-meditators (MD = −13.91 [−23.35; −4.47] mg/dL; *p* = 0.02; 245 subjects). The degree of heterogeneity among the studies was very low (I^2^ = 0.0%). Even after excluding the study at a high risk of bias [46], the overall result remained consistent (MD = −10.88 [−17.50; −4.26] mg/dL; *p* = 0.02).

**Figure 3:**
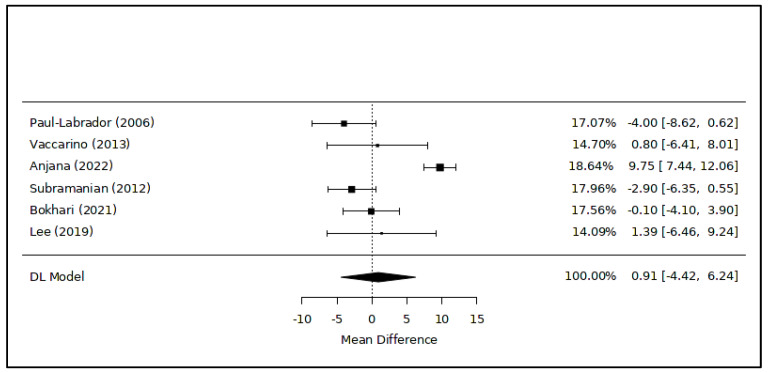
Meta-analysis of variations in HDL cholesterol levels associated with meditation. Figure caption: The first column lists the names of the first authors and the publication dates of the included studies [43,44,45,46,47,48]. The graphical segments represent the effect sizes of individual studies, while the diamond symbolizes the combined results of the quantitative synthesis. The contribution of each study, indicated as its weight, is detailed in the column on the right.

The meta-analysis examining changes in HDL cholesterol levels associated with meditation showed no significant difference between the intervention and control (MD = 0.91 [−4.42; 6.24] mg/dL; *p* = 0.68; 348 subjects) (Figure 3). In total, six RCTs were included in this analysis [43,44,45,46,47,48], and the level of heterogeneity among the studies was substantial (I^2^ = 91.0%). The leave-one-out analysis showed a similar result (MD = 1.74 [−4.82; 8.30] mg/dL; *p* = 0.50).

**Figure 4:**
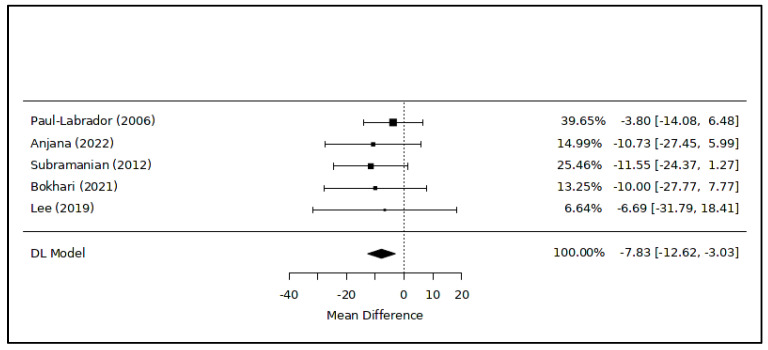
Meta-analysis of variations in LDL cholesterol levels associated with meditation. Figure caption: The first column lists the names of the first authors and the publication dates of the included studies [43,45,46,47,48]. The graphical segments represent the effect sizes of individual studies, while the diamond symbolizes the combined results of the quantitative synthesis. The contribution of each study, indicated as its weight, is detailed in the column on the right.

The meta-analysis examining variations in LDL cholesterol levels associated with meditation included five RCTs [43,45,46,47,48] (Figure 4): the overall result showed a significant difference in favor of the intervention (MD = −7.83 [−12.62; −3.03] mg/dL; *p* = 0.01; 280 subjects). The level of heterogeneity among the studies was extremely low (I^2^ = 0.0%). The leave-one-out analysis excluding the study at a high risk of bias confirmed the overall result (MD = −8.74 [−13.23; −4.25] mg/dL; *p* = 0.01).

**Figure 5:**
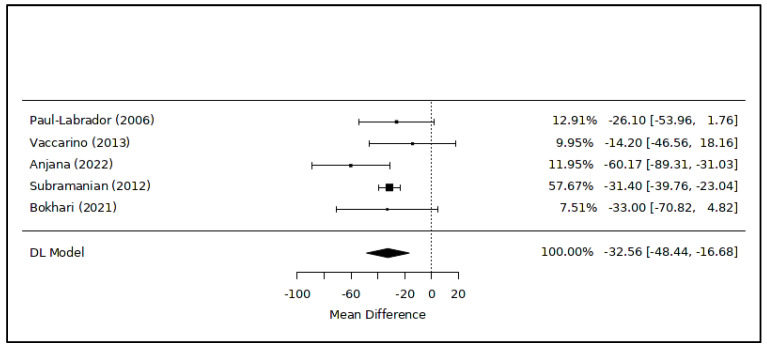
Meta-analysis of variations in triglyceride levels associated with meditation. Figure caption: The first column lists the names of the first authors and the publication dates of the included studies [43,44,45,46,47]. The graphical segments represent the effect sizes of individual studies, while the diamond symbolizes the combined results of the quantitative synthesis. The contribution of each study, indicated as its weight, is detailed in the column on the right.

The meta-analysis examining the modifications in triglyceride levels associated with meditation included five RCTs [43,44,45,46,47] (Figure 5): the overall result showed a significant difference in favor of the intervention (MD = −32.56 [−48.44; −16.68] mg/dL; *p* < 0.01; 313 subjects). The level of heterogeneity among the studies was low (I^2^ = 19.2%). The leave-one-out analysis, which involved excluding the study with a high risk of bias, corroborated the result (MD = −33.92 [−65.77; −2.06] mg/dL; *p* = 0.04).

## 4. Discussion

### 4.1. Brief Summary of the Available Evidence and Potential Explanations

The principal findings of this study suggest an association between meditation practice in healthy or sub-healthy adults and reduced cholesterol levels, with an average decrease of approximately −14 mg/dL (Figure 2), alongside favorable and even more pronounced impacts on triglyceride levels (−33 mg/dL on average, as shown in Figure 5). Additionally, regular engagement in static meditation appears to contribute to lowered total and LDL cholesterol levels, averaging around −8 mg/dL (Figure 4). Furthermore, certain meditation techniques show potential correlations with improved serum concentrations of HDL cholesterol (Table 2).

These observed effects might be attributed to the alterations induced by meditation on autonomic nervous system activity, characterized by an increase in ventral vagal tone and a decrease in adrenergic activity [61,62]. It is noteworthy that autonomic dysregulation, often associated with certain psychiatric disorders, has been linked to dyslipidemia, evidenced by elevated triglyceride levels and, to a lesser extent, altered cholesterolemia [63]. Moreover, stress-induced adrenergic hyperactivation can result in the abnormal and prolonged release of stress hormones such as cortisol, thereby stimulating lipid metabolism and leading to elevated circulating levels of fats [64,65,66]. Consequently, interventions aimed at mitigating the disruption caused by stress- or disease-induced autonomic dysregulation may exert a counteractive influence on serum lipid levels. In fact, data from large cohort studies, analyzing the relationship between performing any mind–body practices and metabolic parameters in middle-aged adults, indicated lower blood lipid concentrations (especially triglycerides) in those who regularly engage in meditative exercises [67]. This underscores the potential for meditation techniques to serve as modulators of autonomic nervous system activity, offering a promising avenue for the integrative management of dyslipidemia and its associated health implications.

Furthermore, certain Eastern meditation techniques exhibit potential associations with enhanced serum concentrations of HDL cholesterol (Table 2), likely attributed to their inclination to promote a more active lifestyle and a healthier diet. This encouragement is probably facilitated through an engagement with nature, vegetarianism, and participation in various physical activities (walking meditation, forest bathing, martial arts) aligned with the basic principles of Eastern philosophies. These observations are in accord with what has been suggested by other authors, who have underscored that the metabolic effects of a multi-component meditation program are significantly influenced by the amount of physical exercise involved in active mind–body practices like qigong [32].

In addition to its potential impact on serum lipid levels, meditation is recognized for its myriad benefits on both mental and physical health outcomes such as stress reduction, improved mental well-being, enhanced focus, and better overall emotional resilience [1,68], even in patients with cardiovascular diseases or risk factors [69,70,71]. Furthermore, the absence of adverse effects associated with meditation enhances its appeal as a complementary approach to traditional interventions. While further research is essential to elucidate causation and determine optimal practices, the current evidence underscores meditation’s potential as a safe and multifaceted strategy for enhancing cardiovascular health and overall quality of life.

### 4.2. Current Guidelines and Practical Implications of the Study Results

The European Society of Cardiology (ESC) guidelines on the management of dyslipidemias highlight the importance of lifestyle modifications in achieving specific treatment goals [72]. These modifications include dietary changes, physical activity, weight management, and other behavioral adjustments that directly influence lipid levels and, consequently, cardiovascular risk. Dietary factors have a significant impact on LDL cholesterol levels: saturated fatty acids elevate LDL cholesterol levels, whereas unsaturated fats from sources like safflower, sunflower, rapeseed, flaxseed, corn, olives, or soybean can reduce LDL cholesterol levels [73]. Bodyweight reduction and regular physical exercise, though having lesser impacts on LDL cholesterol levels compared to diet, contribute beneficially to a cardiovascular risk profile through effects on other risk factors, particularly hypertension and diabetes [74,75]. Weight reduction is advised for improving triglyceride levels and insulin sensitivity, and it favorably influences HDL cholesterol levels. Psychosocial stress has been increasingly recognized as a pivotal risk modifier in the development and progression of atherosclerosis: research demonstrates a dose–response relationship between psychosocial stressors, inclusive of mental disorder symptoms, loneliness, and critical life events, and the escalation of atherosclerotic cardiovascular disease risk, independent of traditional risk factors and irrespective of sex [76]. While psychosocial stress directly influences biological pathways, it also significantly correlates with socioeconomic and behavioral risk elements such as smoking and poor adherence to treatment regimens. The association between psychosocial stress and cardiovascular health highlights the need to consider stress management as an integral component of atherosclerotic cardiovascular disease prevention and treatment strategies [77,78].

The practical implications arising from the main findings of this review for individuals with dyslipidemia involve the integration of meditation into a holistic treatment plan. Trials consistently recommend engaging in meditation for around 15–20 min a day, at least five days a week, as this established routine is linked to positive outcomes. Particularly important are the observed benefits in stress-prone demographics, underscoring meditation’s preventive potential for cardiovascular health. The optimization of integrating meditation into lipid management strategies necessitates a tailored patient education, vigilant monitoring, and proper adjustments. This review positions meditation as a non-invasive and holistic adjunct to traditional interventions, providing a promising avenue for enhancing overall cardiovascular well-being.

### 4.3. Study Limitations

One limitation of this review stems from the inherent heterogeneity across individual studies, including variations in methodologies, sample sizes, and participant characteristics. Publication bias, where studies with positive results are more likely to be published, can affect the comprehensiveness of the analysis. In particular, the assessment of publication bias through funnel plots was precluded due to the limited number of studies eligible for quantitative synthesis (in fact, according to the Cochrane guidelines, a minimum of ten studies is recommended for conducting this analysis [42]). Similarly, due to the same constraints, it was not possible to carry out a meta-regression analysis to examine the potential sources of heterogeneity. However, the search strategy was kept as wide as possible to retrieve all relevant studies and have a picture of the existing evidence. Moreover, the diversity of meditation practices and the limited number of high-quality Randomized Controlled Trials pose challenges to drawing definitive conclusions. As the field evolves, ongoing research with standardized methodologies and diverse participant groups is essential to address these limitations and provide more robust insights into the impact of meditation on blood lipid profiles.

## 5. Conclusions

In summary, regular engagement in static meditation practices can be associated with lower triglyceride and, to a lesser extent, cholesterol levels. In the broader context of preventive measures against cardiovascular diseases, incorporating stress-reducing techniques such as meditation into one’s lifestyle emerges as a valuable component for health promotion. The benefits derived from the modulation of autonomic nervous system activity and the attenuation of stress-related responses position meditation as a promising avenue for individuals seeking to proactively manage their well-being. This recognition underscores the significance of integrating stress management strategies, such as regular meditation practice, into holistic approaches aimed at fostering cardiometabolic health and overall well-being.

Further studies on the topic are advised to deepen our understanding of the relationship between meditation and blood lipid levels: in particular, these investigations could elucidate specific techniques, practice durations, and the demographic factors influencing lipid profile benefits. A better comprehension of these interactions holds the potential to refine preventative strategies and contribute evidence-based recommendations for health promotion.

## Figures and Tables

**Table 1:** Summary of the scientific evidence about the effects of meditation practice on blood lipid levels (cholesterol and triglycerides).

Population Characteristics	Intervention (*n*)	Comparison (*n*)	Outcomes (Mean ± SD)		Study Design	Reference
			Cholesterol [mg/dL]	Triglycerides [mg/dL]		
103 patients with coronary heart disease (age range: 50–80 yo; 84 M—19 F)	Transcendental Meditation every day for 16 weeks, supported by personal lectures and group meetings (*n* = 52)	No meditation, only a health education program (*n* = 51)	TC (INT vs. CON): 158.6 ± 24.2 vs. 167.9 ± 28.6 (ns) HDL-C (INT vs. CON): 44.3 ± 8.3 vs. 48.3 ± 14.7 (ns) LDL-C (INT vs. CON): 89.0 ± 21.5 vs. 92.8 ± 30.8 (ns)	TG (INT vs. CON): 126.7 ± 56.2 vs. 152.8 ± 84.9 (ns)	RCT	[43]
68 patients with risk factors for metabolic syndrome (age range: 30–65 yo; 14 M—54 F)	Consciously resting meditation, a mantra-based meditation (20 min) twice a day for 12 months (*n* = 33)	No meditation, only health education (*n* = 35)	HDL-C (INT vs. CON): 57.1 ± 17.5 vs. 56.3 ± 12.2 (ns)	TG (INT vs. CON): 109.6 ± 36.6 vs. 123.8 ± 90.1 (*)	RCT	[44]
65 patients with hypertension (age range: 25–60 yo; 29 M—36 F)	Om chanting (5 min) + yoga nidra (20 min) 5 days a week for 2 months (*n* = 34)	No meditation (*n* = 31)	TC (INT vs. CON): 237.32 ± 35.07 vs. 250.44 ± 35.50 (*) HDL-C (INT vs. CON): 44.53 ± 3.86 vs. 34.78 ± 5.43 (*) LDL-C (INT vs. CON): 155.93 ± 34.39 vs. 166.66 ± 34.3 (*)	TG (INT vs. CON): 184.83 ± 72.67 vs. 245 ± 45.00 (*)	RCT	[45]
40 students under exam stress (age range: 18–23 yo; 20 M—23 F)	Sudarshan kriya and pranayama meditation (~15 min) every day for 6 weeks (*n* = 21)	No meditation (*n* = 19)	TC (INT vs. CON): 146.7 ± 23.6 vs. 168.2 ± 18.2 (*) HDL-C (INT vs. CON): 42.5 ± 7.6 vs. 45.4 ± 2.6 (ns) LDL-C (INT vs. CON): 90.85 ± 27.0 vs. 102.4 ± 12.4 (ns)	TG (INT vs. CON): 71.0 ± 11.4 vs. 102.4 ± 15.1 (*)	RCT	[46]
37 patients with history of coronary heart disease (age range: 50–80 yo; 22 M—15 F)	Transcendental Meditation twice a week + cardiac rehabilitation program (*n* = 19)	Cardiac rehabilitation program (*n* = 18)	Changes from baseline values after 12 weeks: TC (INT vs. CON): 0.0 ± 37.0 vs. +14.8 ± 34.0 (ns) HDL-C (INT vs. CON): +1.1 ± 6.0 vs. +1.2 ± 6.4 (ns) LDL-C (INT vs. CON): +0.3 ± 27.0 vs. +10.3 ± 28.1 (ns)	Changes from baseline values after 12 weeks: TG (INT vs. CON): −16.4 ± 76.8 vs. +16.6 ± 33.4 (ns)	RCT	[47]
35 patients with hypertension and type 2 diabetes mellitus (age range: 60–80 yo; 24 M—19 F)	Brain Education Sangdahnjeon meditation twice a week for 8 weeks (*n* = 21)	No meditation, only health education advice (*n* = 14)	HDL-C (INT vs. CON): 49.10 ± 13.07 vs. 47.71 ± 10.51 (ns) LDL-C (INT vs. CON): 90.67 ± 35.54 vs. 97.36 ± 38.14 (ns)	N/A	RCT	[48]
76 adults, including 18 smokers, 22 patients with hypertension, and 36 healthy subjects (18 allocated to INT and 18 to CON) (age range: 25–60 yo; 27 M—49 F)	Meditation with biofeedback-aided relaxation (30 min) every day for 6 weeks (*n* = 58)	No meditation (*n* = 18)	TC (INT in patients with hypertension vs. CON): 223 ± 44 vs. 234 ± 49 (*) Other comparisons between groups were not statistically significant.	TG (INT in patients with hypertension vs. CON): 132 ± 65 vs. 96 ± 28 Other comparisons between groups were not statistically significant too.	non-RCT	[49]
30 healthy university students (age range: 17–22 yo; 0 M—30 F)	Transcendental Meditation (20 min) twice a day for 12 weeks (*n* = 15)	Relax with closed eyes (20 min) twice a day for 12 weeks (*n* = 15)	TC (INT: baseline—6 weeks—12 weeks): 162.5 ± 10.4; 161.2 ± 11.14 (*); 159 ± 11.11 (*) Comparisons between INT and CON values were not described.	N/A	non-RCT	[50]
23 patients with hypercholesterolemia (age range: 40–50 yo; 14 M—9 F)	Transcendental Meditation (20 min) twice a day for 13 months (*n* = 12)	No meditation (*n* = 11)	TC (INT vs. CON): 225.0 ± 9.4 vs. 254.0 ± 11.3 (*)	N/A	non-RCT	[51]
17 healthy subjects, including 10 experienced meditators (age range: 30–50 yo; 5 M—12 F)	Zen meditation (1.5 h) five days per week for 6 weeks (*n* = 7)	No meditation (*n* = 10)	HDL-C (INT) from 45.2 ± 8.9 to 53.0 ± 12.8 (*)	TG (INT): from 152.3 ± 125.8 to 221.4 ± 163.9 (ns)	non-RCT	[32]
76 adults, including 61 healthy subjects and 15 patients with depression (age range: 18–65 yo; M/F ?)	Mindfulness-based meditation (20 min) every day for 2 months (*n* = 76)	N/A	TC (pre–post): significant reduction in healthy subjects (*), but not in patients with depression (ns). The same was observed for HDL-C levels (data were only visually displayed).	TG (pre–post): significant reduction in both healthy subjects (*) and patients with depression (*) (data were only visually displayed).	Pre–post study	[52]
252 subjects from Sri Lanka, including 151 Buddhist monks and 101 laymen (age range: 40–60 yo; 252 M—0 F)	Buddhist monks meditating for >6 months according to the principles of Samatha and Vipassana meditation (*n* = 71) (INT-1) Buddhist monks meditating for <6 months (*n* = 44) (INT-2)	Buddhist monks not engaged in regular meditation (*n* = 36) (CON-1) Laymen without experience in meditation (*n* = 101) (CON-2)	TC (INT-1 vs. INT-2 vs. CON-1 vs. CON-2): 172.5 ± 23.4 vs. 173.3 ± 34.6 vs. 187.8 ± 45.2 vs. 174.6 ± 48.5 (ns) HDL-C (INT-1 vs. INT-2 vs. CON-1 vs. CON-2): 53.8 ± 5.3 (*) vs. 45.6 ± 5.8 vs. 42.8 ± 5.4 vs. 39.1 ± 7.4 LDL-C (INT-1 vs. INT-2 vs. CON-1 vs. CON-2): 93.26 ± 22.5 vs. 101.0 ± 32.4 vs. 114.9 ± 27.8 vs. 119.7 ± 34.9 (ns)	TG (INT-1 vs. INT-2 vs. CON-1 vs. CON-2): 136.5 ± 36.4 vs. 141.8 ± 42.4 vs. 126.1 ± 37.1 vs. 158.9 ± 56.7 (ns)	OS (case–control)	[53]
106 Tibetan healthy subjects (age range: 30–50 yo; 106 M—0 F)	Tibetan monks of 3 sects (Gelug, Nyingma, Sakya) with at least 5 years of experience in meditation practice (*n* = 48)	Healthy controls with no experience in meditation and the same dietary habits as monks (*n* = 37)	TC Gelug (*n* = 29) vs. CON (*n* = 17): 185.2 ± 40.6 vs. 181.4 ± 38.3 (ns) Nyingma (*n* = 9) vs. CON (*n* = 10): 180.2 ± 46.8 vs. 207.7 ± 32.1 (*) Sakya (*n* = 10) vs. CON (*n* = 10): 192.2 ± 33.3 vs. 207.7 ± 32.1 (ns) LDL-C Gelug vs. CON: 120.7 ± 39.4 vs. 111.8 ± 33.3 (ns) Nyingma vs. CON: 114.1 ± 35.6 vs. 138.8 ± 31.3 (*) Sakya vs. CON: 119.1 ± 25.5 vs. 138.8 ± 31.3 (*)	N/A	OS (case–control)	[54]
105 adult volunteers (age range: 30–70 yo; 42 M—63 F)	>5 years of experience in Raja Yoga meditation (INT-1) Up to 5 years of experience in Raja Yoga meditation (INT-2)	No experience in meditation (CON)	TC (CON vs. INT-2 vs. INT-1): 293.0 ± 67.9 vs. 240.6 ± 69.1 (*) vs. 235.4 ± 94.5 (*) HDL-C (CON vs. INT-2 vs. INT-1, median (min; max)): 46 (24; 120) vs. 44 (24; 99) vs. 52 (38; 76) (ns)	TG (CON vs. INT-2 vs. INT-1, median (min; max)): 141.5 (64.2; 435.3) vs. 105.0 (19.0; 409.4) vs. 123.0 (51.0; 212.6) (ns)	OS (case–control)	[55]
65 women with menopausal symptoms (age range: 37–56 yo; 0 M—65 F)	Brain Education Sangdahnjeon meditation at least once a week for 6 months (*n* = 33)	No experience in meditation (*n* = 32)	Postmenopausal period: HDL-C (INT vs. CON): 65.87 ± 12.95 vs. 58.07 ± 13.53 Significant interaction between group and (pre–post) menopausal state (*)	Perimenopausal period: TG (INT vs. CON): 86.86 ± 43.30 vs. 104.42 ± 63.90 (ns)	OS (case–control)	[56]
49 healthy women (age range: ?; 0 M—49 F)	Pre-(*n* = 8) and post-(*n* = 9) menopausal women with >5 years of experience in Raja Yoga meditation (INT-1) Pre-(*n* = 8) and post-(*n* = 9) menopausal women with up to 5 years of experience in Raja Yoga meditation (INT-2)	Pre- (*n* = 7) and post- (*n* = 8) menopausal women without experience in meditation (CON)	Premenopausal period: TC (CON vs. INT-2 vs. INT-1): 235.0 ± 61.2 vs. 202.5 ± 33.5 vs. 189.4 ± 47.6 (ns) HDL-C (CON vs. INT-2 vs. INT-1): 50.0 ± 26.8 vs. 42.5 ± 5.4 vs. 54.7 ± 11.7 (ns) LDL-C (CON vs. INT-2 vs. INT-1): 164.5 ± 41.4 vs. 137.0 ± 40.6 vs. 112.7 ± 47.0 (ns) Postmenopausal period: TC (CON vs. INT-2 vs. INT-1): 332.3 ± 74.5 vs. 238.8 ± 65.0 (*) vs. 232.2 ± 38.5 (*) HDL-C (CON vs. INT-2 vs. INT-1): 60.5 ± 29.6 vs. 56.6 ± 21.4 vs. 52.3 ± 12.8 (ns) LDL-C (CON vs. INT-2 vs. INT-1): 235.4 ± 68.0 vs. 149.6 ± 79.2 (*) vs. 151.3 ± 37.4 (*)	Premenopausal period: TG (CON vs. INT-2 vs. INT-1): 102.1 ± 43.4 vs. 114.8 ± 55.7 vs. 109.9 ± 40.2 (ns) Post-menopausal period: TG (CON vs. INT-2 vs. INT-1): 181.8 ± 86.0 vs. 163.3 ± 110.1 vs. 143.1 ± 42.0 (ns)	OS (case–control)	[57]

Table description: studies eligible for inclusion in this review are grouped on the basis of their design and, within each group, they are listed according to the sample size in descending order. * = statistically significant difference between intervention and control in favor of the intervention (*p* < 0.05). CON = Control/Comparison Group; F = Females; HDL-C = High-Density Lipoprotein Cholesterol Levels; INT = Intervention Group; IQR = Interquartile Range; LDL-C = Low-Density Lipoprotein Cholesterol Levels; M = Males; N/A = Not Available; non-RCT = Non-Randomized Controlled Trial; ns = not statistically significant (*p* ≥ 0.05); OS = Observational Study; RCT = Randomized Controlled Trial; SD = Standard Deviation; TC = Total Cholesterol Levels; TG = Triglyceride Levels; yo = years old.

**Table 2:** Summary of study outcomes: effects of static meditation techniques on blood lipid levels.

Meditation Technique and Duration of Study			Sample Size	CV Risk Factors	TC	HDL-C	LDL-C	TG	Study Design	Ref.
Ayurvedic-based meditation techniques	Transcendental Meditation	16 weeks	103	Yes	=	=	=	=	++++	[43]
		12 weeks	37	Yes	=	=	=	=	++++	[47]
		12 weeks	30	No	↓	N/A	N/A	N/A	+++	[50]
		13 months	23	Yes	↓	N/A	N/A	N/A	+++	[51]
	Yoga-based meditation techniques (without body movements)	2 months	65	Yes	↓	↑	↓	↓	++++	[45]
		6 weeks	40	No	↓	=	=	↓	++++	[46]
		-	105	No	↓	=	N/A	=	+	[55]
		-	49	No	↓	=	↓	=	+	[57]
Mindfulness-based meditation techniques	Mindfulness or consciously resting meditation	12 months	68	Yes	N/A	=	N/A	↓	++++	[44]
		6 weeks	76	Yes	↓	N/A	N/A	=	+++	[49]
		2 months	76	No	↓	↓	N/A	↓	++	[52]
Eastern meditation techniques with spiritual origin	Brain Education Sangdahnjeon meditation	8 weeks	35	Yes	N/A	=	=	N/A	++++	[48]
		-	65	No	N/A	↑	N/A	=	+	[56]
	Spiritual (Zen, Buddhist, Tibetan) meditation	6 weeks	17	No	N/A	↑	N/A	=	+++	[32]
		-	252	?	=	↑	=	=	+	[53]
		-	106	No	↓	N/A	↓	N/A	+	[54]

Table description: studies eligible for inclusion in this review are grouped on the basis of the meditation technique. ↓: Statistically significant decrease (*p* < 0.05) in blood lipid levels (post-test intervention versus control group values in controlled trials or pre–post test difference in uncontrolled studies); ↑: Statistically significant increase (*p* < 0.05) in blood lipid levels. =: No statistically significant (*p* ≥ 0.05) modification of blood lipid levels; CV risk factors (Yes/No/?) = cardiovascular risk factors (dyslipidemia, diabetes, hypertension, etc.); HDL-C = High-Density Lipoprotein Cholesterol Levels; LDL-C = Low-Density Lipoprotein Cholesterol Levels; N/A = Not Available; Ref. = reference/citation. Study design: ++++ = Randomized Controlled Trial; +++ = Non-Randomized Controlled Trial; ++ =Pre–Post Study; + = Observational Study; TC = Total Cholesterol Levels; TG = Triglyceride Levels.

**Table 3:** Quality assessment of the RCTs included in this review.

First Author (Date of Publication)	1	2	3	4	5	6	7	Overall	Ref.
Paul-Labrador (2006)	(+)	(+)	/	/	(+)	(+)	(+)	(+)	[43]
Vaccarino (2013)	(+)	(+)	/	/	(+)	(+)	(+)	(+)	[44]
Anjana (2022)	(+)	(?)	/	/	(+)	(+)	(+)	(?)	[45]
Subramanian (2012)	(?)	(?)	/	/	(+)	(+)	(+)	(−)	[46]
Bokhari (2021)	(+)	(+)	/	/	(+)	(+)	(+)	(+)	[47]
Lee (2019)	(+)	(+)	/	/	(?)	(+)	(+)	(?)	[48]

Table legends: Ref. = reference/citation; Risk of bias: (+) = low risk of bias; (?) = unclear risk of bias; (−) = high risk of bias. Domains: 1. Random sequence generation; 2. Allocation concealment; 3. Blinding of participants; 4. Blinding of outcome assessment; 5. Incomplete outcome data; 6. Selective reporting; 7. Other bias.

**Table 4:** Quality assessment of the studies other than RCTs included in this review.

First Author (Date of Publication)	1	2	3	4	5	6	7	8	9	10	11	12	13	14	Study Design	Ref.
Patel (1977)	N	/	?	/	/	Y	Y	Y	Y	Y	Y	?	?	Y	non-RCT	[49]
Bhatnagar (2015)	N	/	?	/	/	?	Y	Y	Y	Y	Y	?	?	Y		[50]
Cooper (1979)	N	/	?	/	/	Y	Y	Y	Y	Y	Y	?	?	Y		[51]
Kormanovski (2008)	N	/	?	/	/	Y	Y	Y	Y	Y	Y	?	?	Y		[32]
Xue (2018)	Y	Y	Y	Y	?	Y	Y	/	N	Y	N	/	/	/	Pre–post study	[52]
Kumbukgolla (2019)	Y	Y	N	Y	?	Y	N	?	Y	Y	/	Y	/	/	OS (case–control)	[53]
Xue (2022)	Y	Y	?	Y	Y	Y	N	Y	Y	Y	/	Y	/	/		[54]
Vyas (2002)	Y	?	N	Y	Y	Y	?	?	?	?	/	Y	/	/		[55]
Sung (2020)	Y	Y	Y	Y	Y	Y	?	Y	Y	Y	/	Y	/	/		[56]
Vyas (2008)	Y	?	N	Y	Y	Y	?	?	?	?	/	Y	/	/		[57]

Table legends: Ref. = reference/citation; Y = yes; N = no; ? = cannot be determined; / = not applicable. For a comprehensive description of the domains covered by the quality assessment tools, it is possible to consult the NIH website at https://www.nhlbi.nih.gov/health-topics/study-quality-assessment-tools (accessed on 9 March 2024).

## Data Availability

Data sharing not applicable.
